# Handbook of Vertebrate Dissection

**Published:** 1884-08

**Authors:** 


HANDBOOK OF VERTEBRATE DISSECTION. PART III. *How to Dissect a Rodent. By* H. NEWELL MARTIN, D. SC., M. D., M. A., *Professor in the Johns Hopkins University, and* WILLIAM A. MOALE, M. D. *12mo., pp. 247. New York:* MACMILLAN & CO. *1884.*

This little book fulfills, with unusual freedom from fault, the function for which it was intended. As a *handbook* for use at the dissecting-table, it is a valuable acquisition.

It is a matter of regret with us, however, that the rabbit was not chosen instead of the rat. The former is the favorite animal in Germany, is peculiarly adapted to the purposes of physiological investigation, and is quite as abundant and widely distributed.

The text is remarkably free from technical and typographical errors.

We commend the book most heartily to our readers.

